# Staged heterogeneity learning to identify conformational B-cell epitopes from antigen sequences

**DOI:** 10.1186/s12864-017-3493-0

**Published:** 2017-03-14

**Authors:** Jing Ren, Jiangning Song, John Ellis, Jinyan Li

**Affiliations:** 1 Advanced Analytics Institute, Faculty of Engineering and Information Technology, University of Technology Sydney, Ultimo, NSW 2007 Australia; 2 College of Computer, National University of Defense Technology, Changsha, 410073 China; 3 Monash Centre for Data Science, Faculty of Information Technology, Monash University, Melbourne, VIC 3800 Australia; 4 Infection and Immunity Program, Biomedicine Discovery Institute, Monash University, Melbourne, VIC 3800 Australia; 5 School of Life Sciences, University of Technology Sydney, Ultimo, NSW 2007 Australia; 6 Advanced Analytics Institute and Centre for Health Technologies, Faculty of Engineering and Information Technology, University of Technology Sydney, Ultimo, NSW 2007 Australia

**Keywords:** Staged heterogeneity learning, Conformational epitope, B-cell epitope, Epitope prediction, Sequence-based

## Abstract

**Background:**

The broad heterogeneity of antigen-antibody interactions brings tremendous challenges to the design of a widely applicable learning algorithm to identify conformational B-cell epitopes. Besides the intrinsic heterogeneity introduced by diverse species, extra heterogeneity can also be introduced by various data sources, adding another layer of complexity and further confounding the research.

**Results:**

This work proposed a staged heterogeneity learning method, which learns both characteristics and heterogeneity of data in a phased manner. The method was applied to identify antigenic residues of heterogenous conformational B-cell epitopes based on antigen sequences. In the first stage, the model learns the general epitope patterns of each kind of propensity from a large data set containing *computationally defined* epitopes. In the second stage, the model learns the heterogenous complementarity of these propensities from a relatively small guided data set containing *experimentally determined* epitopes. Moreover, we designed an algorithm to cluster the predicted individual antigenic residues into conformational B-cell epitopes so as to provide strong potential for real-world applications, such as vaccine development. With heterogeneity well learnt, the transferability of the prediction model was remarkably improved to handle new data with a high level of heterogeneity. The model has been tested on two data sets with experimentally determined epitopes, and on a data set with computationally defined epitopes. This proposed sequence-based method achieved outstanding performance - about twice that of existing methods, including the sequence-based predictor CBTOPE and three other structure-based predictors.

**Conclusions:**

The proposed method uses only antigen sequence information, and thus has much broader applications.

## Background

B-cell immunity provides a natural barrier for a host to block the invasion of pathogens into cells. A vital medium of this mechanism is the B-cell epitope, a small surface area of an antigen that can be recognized and bound by an antibody. The majority (more than 90%) of B-cell epitopes are conformational epitopes which are compact in 3D space but not continuous in sequence [1]. B-cell epitopes are able to stimulate B-cells to produce neutralizing antibodies, and can be used to design safe vaccines, especially for vulnerable populations such as infants, young children and the elderly, to be immunized against infectious diseases [2]. Their accurate prediction is thus of great significance, however, inhibited by several unsolved issues. One serious issue is the broad heterogeneity of epitope data.

**Fig. 1:**
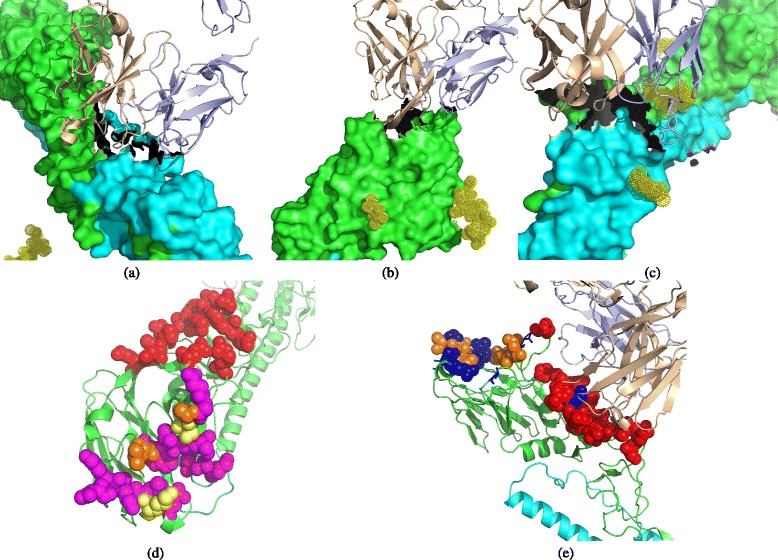
Diversity of B-cell epitopes. (**a**) H2-C179, (**b**) H3-C05, (**c**) H3-CR8043, (**d**) A/Viet Nam/1203/2004(H5N1), (**e**) A/X-31(H3N2) **a**–**c** show diversified antigen-antibody interactions. Antigens are shown in surface, and antibodies are shown in cartoon (heavy chain: *wheat color*, light chain: *light blue*). **d** and **e** illustrate the diversity induced by assay types. The epitopes determined by crystallography are colored in *red* and *magenta*, the epitopes determined by ELISA are colored in *orange* and *yellow*, and the epitopes determined by biological activity are colored in *blue*

Intrinsic heterogeneity exists in antigen-antibody interactions due to long time evolution and frequent mutation of pathogens, resulting in much non-trivial variance in binding shapes and amino acid propensities. For example, antibody Fab C179 binds to a *concave* region of the H2 hemagglutinin, through a paratope mainly composed of *loops* of the heavy and light chains (Fig. 1 a). Differently, antibody C05 binds to a *protrusive* region of the H3 hemagglutinin via a *sheet* segment of its heavy chain (Fig. 1 b). A more complicated example is the antibody CR8043, which binds to a *protrusive* region of the H3 hemagglutinin by using segments of several *sheets* in the heavy chain together with *loops* in the light chain (Fig. 1 c).

Technique-induced heterogeneity adds another layer of complexity. In fact, assay difference has a strong influence on the annotation of conformational epitopes. Figure 1 (d–e) illustrates the heterogeneity introduced by wet-lab experiments. In (d) and (e), the epitopes determined by crystallography technologies (represented by the red and magenta spheres) usually cover all the antigenic residues bound by a specific antibody. Though the epitopes determined by an ELISA assay (the orange and yellow spheres) or a biological activity assay (colored in blue) could sometimes be only parts of a conformational epitope, these residues are functionally important and are likely to play a vital role in antigen-antibody binding activity.

Such high levels of heterogeneity in B-cell epitope data sets brings huge challenges for data mining algorithms when attempting to make accurate predictions of the vast number of unknown epitopes. Some previous studies have even yielded conflicting conclusions partially due to insufficient understanding of this highly complex issue. For example, Thornton et al. claimed that continuous epitopes are usually located in protruding regions of proteins [3]. This hypothesis was supported by [4], and the protrusion index was followed to identify conformational epitopes. Conversely, Kringelum et al. conjectured that epitopes should be located in flatten areas, based on their analysis of 107 antigen-antibody bound structures [5]. Hydrophilicity is another widely applied propensity in epitope prediction. In [6], it was confirmed on 92 unbound structures that hydrophilic residues can occur significantly more frequently in epitopes than in other surface areas, while hydrophobic residues can be depleted. Yet, Kringelum et al. found that there is no significant deviation in amino acid preference between epitopes and non-epitope antigen surfaces [5]. Brief consideration of the heterogeneity issue has come to the fore recently. Qi et al. took the immune host information into account and constructed a prediction server, SEPPA 2.0 [7]. We performed propensity analyses on several major antigen types (e.g., virus and bacterial) [6]. As these methods were based on the knowledge of species, their training and analyses were limited by data scale; for minority species, under-fitting is a difficult issue for them to deal with.

Most of the current well-performing prediction methods are structure-based methods [1, 4, 7–15]. They often use structural information like ASA [8], protrusion index [4], contact number [1] and secondary structure [13] to achieve a higher prediction accuracy. However, a major drawback of structure-based methods is the relatively small number of available protein structures. It severely limits their application scope. In PDB (Protein Data Bank), only 115764 biological macromolecular structures were released between 1976 and 4 Feb 2016, some of which are of poor quality (e.g., low resolution). However, a much larger number of antigen sequences have been or can be translated from DNA sequences with ease. Thus, sequence-based prediction methods, if they can match or enhance the performance of structure-based methods, will greatly improve prediction methodologies.

CBTOPE was the first comprehensive method proposed to predict conformational B-cell epitopes from antigen sequences [16]. Subsequent studies used different combinations of sequence-derived propensities (like amino acid scales and evolutionary propensity) through various data mining methods, including: a weighted linear function [17], a re-sampling and propensity voting method [18], an SVM model BEEPro [19], and a cost-sensitive ensemble learning method CBEP [20]. These methods handled neither the heterogeneity issue nor the heterogeneity-induced inconsistency in the data well. In most cases, homologous data sets or a single data set was used to train their models, only capturing general epitope patterns. For example, CBTOPE was trained on an in-house data set extracted from IEDB [16], and [18] used a bound-structure data set and an unbound data set to train two separate models. These methods are likely to have reduced performance on heterologous data sets.

In this paper, we design a new model for **Se**quence-based conformational B-cell epitope **Pre**diction (SePre). To address the issue of data heterogeneity, a staged heterogeneity learning method is proposed to identify antigenic residues. In the first stage, four sub-classifiers are constructed using four types of propensity separately. The aim is to learn the epitope patterns of each type of propensity. To identify the intrinsic epitope patterns from the diversified data is a nontrivial task, and therefore requires a large set of training data. In this work, antigen sequences of 190 bound structures with computationally defined epitopes were used. In the second stage, a decision tree model is trained using antigen sequences with diversified experimentally determined epitopes. The aim is to learn the heterogenous complementarity of the propensities to form the basis of antibody-antigen interactions. This is a relative simple process and could be achieved on less guided data. It has the potential to act as a fine tuning tool for minority class prediction. Furnished with the well-learnt heterogeneity, the transferability of this prediction model is remarkably improved to properly handle heterogeneous test data. In addition, a clustering algorithm is also developed to group nearby individual antigenic residues for the recommendation of conformational epitopes.

Our prediction model has demonstrated outstanding performance on two data sets containing experimentally determined epitopes, and on an unbound data set containing computationally defined epitopes. Compared to the best performances of the sequence-based predictor CBTOPE, and three other structure-based predictors, DiscoTope 2.0, ElliPro and SEPPA 2.0, our method performed around twice as well.

The proposed method SePre uses only antigen sequence information. It recommends conformational B-cell epitopes by applying the distance-based clustering algorithm on a structure that is predicted from the given antigen sequence. Thus, it is suitable for large-scale predictions and has much broader applications, such as the discovery of new epitopes and their corresponding antibodies, and the investigation of new antigens of a pathogen.

## Methods

**Fig. 2:**
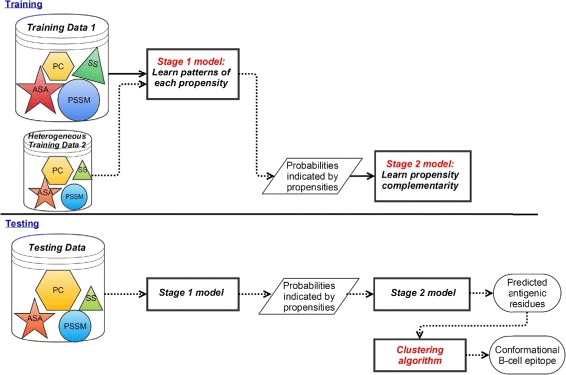
Training and testing processes of the proposed method. The upper part illustrates the training process. The first-stage and second-stage staged heterogeneity learning models are trained on two data sets with heterogenous annotations. The lower part illustrates the testing process of the proposed method. An unsupervised clustering algorithm is involved in the testing process to recommend conformational B-cell epitopes. The shapes inside data sets denote patterns of propensities: PC–physico-chemical propensity, ASA–accessible surface area, SS–secondary structure, PSSM–position-specific scoring matrix

Figure 2 illustrates the training (learning) and testing (prediction) processes of the proposed method. The first-stage and second-stage staged heterogeneity learning models are trained on two data sets with heterogenous annotations. For testing, these two models are used to predict antigenic residues, and then an unsupervised clustering algorithm is deployed to cluster predicted antigenic residues into conformational B-cell epitopes. This section presents details of the heterogeneity learning method, the clustering algorithm for recommending conformational epitopes, and in-house baseline algorithms for performance comparison, as well as the data sets and propensities used.

### Staged heterogeneity learning

The staged heterogenous learning method has two stages of learning (refer to the upper part of Fig. 2). In the first stage, the method learns the general epitope patterns of each type of propensity from a large training data set. For each type of propensity, a sub-classifier is constructed. In the second stage, the method focuses on learning the complementarity of the propensities from a small heterogeneous training data set, taken as guided data. To prepare the training input data for the second stage (shown by dotted arrows), predictions are made by the sub-classifiers on the guided heterogeneous data set. The probabilities predicted by each sub-classifier are used to train the second-stage model. As to the machine learning strategy, random forest is used to train the first-stage sub-classifiers, and decision tree is applied in the second stage.

The lower part of Fig. 2 represents how the proposed method makes predictions. The testing data is firstly predicted by the first-stage sub-classifiers, and the probabilities of the residues belonging to epitopes are generated by each of the sub-classifiers. Then, the second-stage model integrates these probabilities to predict antigenic residues.

The epitope patterns are diversified and relatively complicated to learn, and hence more data is needed for the first-stage learning. However, the second-stage model only needs to deal with a few probability values. A simple learning algorithm and a relative small amount of guided data can be qualified to get good prediction performance.

### Clustering antigenic residues into conformational B-cell epitopes

The staged heterogeneity learning method presented in “Staged heterogeneity learning” section only predicts whether or not a residue is antigenic. It does not distinguish which antigenic residues can be grouped to constitute a conformational B-cell epitope. We propose a clustering method (Algorithm 1) to group the neighboring individual residues into clusters, and recognize each cluster as a conformational B-cell epitope. The aggregated antigenic residues are then recommended with higher priority based on the idea that aggregative antigenic residues are more likely to constitute epitopes [21].



The first step is to search or construct the corresponding antigen structure from an antigen sequence. This involves aligning the target antigen sequence with PDB structures by BLAST. If there is a structure with 100% sequence similarity, it is assigned as the structure of the antigen. If no eligible structure is available, a structure is constructed by I-TASSER [22]. The second step is to cluster the predicted candidate antigenic residues according to their distance information in the structure.

### Data sets

Two types of epitope data sets, extracted from different data sources, were used for the training and assessment of the proposed method: (i) computational epitopes derived from antigen-antibody bound structures in PDB, and (ii) experimentally determined epitopes by various types of assay from IEDB [23]. Two data sets were used in the training: a computational epitope data set named ‘Train190’ was used for training the first-stage model; a small experimental epitope data set named ‘Liang19unbound’ was used as the guided data for training the second-stage model. Three test data sets were used to assess the method’s prediction performance: ‘Exp104’ and ‘Exp163’ contain experimentally determined epitopes, and the third, ‘Ren92unbound’, contains computational defined epitopes.

In this work, a residue is computationally defined as an epitope residue if it has a non-hydrogen atom within 5Å distance from any antibody atom, and loses more than 0.6Å ^2^ of its exposed area upon binding.

#### Details of the two training data sets

‘Train190’ consists of 190 bound structures with 195 non-redundant antigen chains. The epitope annotations were calculated from the antigen-antibody bound structures in PDB. This data set was used for learning the complicated epitope patterns of each kind of propensity in the first stage. It was constructed using the following steps. Firstly, we computationally defined epitopes from 598 antigen-antibody bound structures [6]. Then, the antigen chains were grouped into 217 clusters using CD-HIT [24] with a sequence similarity threshold of 70% (-c 0.7). Next, the epitopes within each cluster were mapped onto a representative chain. In the last step, we removed epitopes that could not be completely aligned with the representative chain and representatives without any epitopes.

‘Liang19unbound’ contains 19 antigen unbound structures. Their epitopes were annotated using IEDB [13]. This data set was used as the guided data to learn the complementarity of propensities in the second stage.

#### Details of the three test data sets

‘Exp104’ contains 104 bound structures with experimentally determined epitopes from IEDB. It was constructed via these steps: (i) extract experimentally determined conformational epitopes from IEDB; (ii) retrieve their source antigen sequences through GeneBank ID, and their corresponding structures through UniProt ID; and (iii) map the epitopes onto these structures.

‘Exp163’ consists of 163 unbound structures whose annotations also came from the experimentally determined epitopes in IEDB. It was used as another test set to assess performance on heterogeneous data with more unbalanced labels.

‘Ren92unbound’ has 92 unbound structures with computational B-cell epitopes [6], and was used to assess the performance of the staged heterogeneity learning method in computational epitopes, as well as the impact of the second-stage heterogeneity learning on the prediction performance in homologous computational epitopes.

### Propensities

Previous studies have shown that epitope residues have a preference on certain propensities. In PUPre [6], we found that even different species demonstrate similar, though not identical, tendencies in propensities such as ASA, RSA, protrusion index and B-factor. Therefore, learning the epitope preferences of propensities is both useful and necessary to heterogeneity learning.

Many propensities are believed to be able to help distinguish epitope residues from non-epitope residues, and have been widely applied in the analysis and prediction of conformational B-cell epitopes, including hydrophilicity [25], amino acid composition [1, 14], conservation score [14, 26], PSSM [17, 19], secondary structure [13], surface exposure propensities [8, 9, 15, 21], contact number [1, 13], and protrusion index [4].

In this paper, we assessed five types of sequence-derived propensities, including: 205 physico-chemical propensities extracted from AAindex [27] with less than 80% similarity, evolutionary propensities–PSSM and conservation score, and predicted structural propensities–ASA and secondary structure. Among them, the physico-chemical propensities were directly extracted from antigen sequences; the PSSM profile and conservation score were generated by PSI-BLAST and ConSurf [28] respectively. The ASA and secondary structure were predicted by SABLE [29]. We found that the conservation score always gave poor performance on epitope identification and sometimes was not available. This is further discussed in “The first-stage performance by single propensities” section. Therefore, it was excluded from the construction of the learning model. To account for the impact of surrounding residues, a seven-residue sliding window was applied to the construction of feature space.

### Baseline algorithms

We applied several baseline algorithms to examine the complementarity of the propensities, including ranked voting, exhaustive voting, and traditional machine learning methods (including SVM, random forest, regression tree, Naïve Bayesian, and Bayesian network). These traditional machine learning methods were implemented by R packages with parameter optimization. Here, we briefly describe the ranked and exhaustive voting algorithms.



Using the ranked voting algorithm (Algorithm 2), propensities are firstly ranked by their performance in identifying conformational B-cell epitopes, and then the selected propensities vote to decide the label of a residue. A residue is predicted as positive if the number of positive votes are greater than or equal to the negative votes (condition 1), and it has at least one positive vote (condition 2). The second condition handles rare situations in which none of the propensities has a positive value. In this case, a negative (silent) prediction is assigned.

Simple combinations of top-ranked propensities do not necessarily guarantee the best prediction performance [1]. Hence, an exhaustive voting algorithm was designed and conducted on all the possible combinations of propensities. Given the original 209 propensities would produce 2^209^−1 combinations, leading to very high complexity for exhaustive voting, we firstly combined the 205 physico-chemical propensities from AAIndex to construct one sub-classifier, and then conducted exhaustive voting on the five groups of propensities.

## Results

This section presents the outstanding epitope prediction performance by our staged heterogeneity learning method in comparison with state-of-the-art methods. Two case studies are also presented to illustrate the prediction details of the heterogeneity learning method.

### Superior performance by staged heterogeneity learning

Our staged heterogeneity learning method, SePre, was tested on both heterogeneous (‘Exp104’ and ‘Exp163’) and homologous (‘Ren92unbound’) data sets. To benchmark the performance, our method was compared with CBTOPE, a sequence-based conformational B-cell epitope predictor [16]. We attempted to compare with other sequence-based prediction methods [17, 18, 20], but their server or software package was not available. We also compared SePre with three typical structure-based predictors: DiscoTope 2.0 [15], ElliPro [4] and SEPPA 2.0 [7]. Their servers provided batch query entries - convenient for large-scale comparison.

The prediction results are summarized in Table 1. On all of the test data sets, SePre had the best F-scores and the best precisions. The best recalls were achieved by ElliPro, but it had a much lower precisions. Overall, SePre achieved significantly better performance than the state-of-the-art methods.

**Table 1:** Prediction performance by staged heterogeneity learning in comparison with other prediction methods

Predictor	Dataset	Recall	Precision	F-score
**SePre**	Exp104	0.454	**0.234**	**0.308**
CBTOPE	Exp104	0.516	0.051	0.092
DiscoTope 2.0	Exp104	0.230	0.066	0.102
ElliPro	Exp104	**0.705**	0.066	0.121
SEPPA 2.0	Exp104	0.524	0.096	0.163
**SePre**	Exp163	0.314	**0.159**	**0.211**
CBTOPE	Exp163	0.492	0.052	0.094
DiscoTope 2.0	Exp163	0.231	0.051	0.083
ElliPro	Exp163	**0.686**	0.047	0.089
SEPPA 2.0	Exp163	0.429	0.063	0.110
**SePre**	Ren92unbound	0.639	**0.426**	**0.511**
CBTOPE	Ren92unbound	0.544	0.133	0.213
DiscoTope 2.0	Ren92unbound	0.279	0.162	0.205
ElliPro	Ren92unbound	**0.702**	0.120	0.205
SEPPA 2.0	Ren92unbound	0.484	0.164	0.245

The boldface data highlights the optimal performance

On the two heterogeneous data sets, the comparing methods demonstrated very low performance: very low precisions in particular. This is probably because the epitopes of the two test data sets are quite different from those of the training data sets. Our prediction performance was as expected, as our method is a heterogeneity-focused learning method. Against ‘Exp104’, SePre had a much higher precision, at 0.234, and achieved a much higher F-score, at 0.308. For a close comparison, although CBTOPE had a slightly higher recall, its F-score was only 0.092. SEPPA 2.0 used antigen structure information in this bound data set and showed the best performance among the three structure-based predictors, but its F-score was only 0.163. On ‘Exp163’, an unbound and more unbalanced data set, SePre still showed a good performance: its F-score was 0.211 with a recall of 0.314 and a precision of 0.159 - nearly twice the performance of the next best predictor, SEPPA 2.0, with an F-score of 0.110. On the homologous data set ‘Ren92unbound’, SePre achieved an excellent F-score of 0.511, while the best F-score of the other predictors was only 0.245, again by SEPPA 2.0. These results imply that our heterogeneity learning method is quite compatible with homologous data as well.

### Prediction results by straightforward staged learning

In conventional practice, a training process is usually conducted on a single homologous data set. To examine the improvement made by staged heterogeneity learning, a straightforward staged learning model (denoted by SePre.v _0_) was also constructed. It has a similar process as SePre, except that its second-stage model is trained on the homologous data from ‘Train190’, and its training input is generated from the LOOCV results of the first-stage model.

Table 2 shows the prediction results using a straightforward staged learning model. The performance of SePre.v _0_ was much lower, compared to SePre. On the two heterogeneous data sets, its precision was also much lower, leading to a considerable decline in F-scores: from 0.308 to 0.153 on ‘Exp104’, and from 0.211 down to 0.102 on ‘Exp163’. Though their recalls improved to 0.793 and 0.744 respectively, these results mean that the predictor has predicted most of the antigen sequences as antigenic residues. Even on the homologous data set ‘Ren92unbound’, SePre.v _0_ suffered from a decrease in F-scores - from 0.511 to 0.310. Over-fitting in the second-stage of training is a possible explanation.

**Table 2:** Prediction results by straightforward staged learning

Predictor	Dataset	Recall	Precision	F-score
SePre.v _0_	Exp104	0.793	0.085	0.153
SePre.v _0_	Exp163	0.744	0.055	0.102
SePre.v _0_	Ren92unbound	0.923	0.186	0.310

SePre.v _0_ in this table is different from the proposed SePre model. Its second-stage model was trained on the leave-one-out cross-validation (LOOCV) results from the first-stage model. In LOOCV, if an antigen has multiple chains, these chains are all left out as tests in a round to reduce the coupling between a target antigen and the training data

### Results on recommending conformational B-cell epitopes

SePre only makes predictions on whether a residue of an antigen sequence is an antigenic residue. Algorithm 1 can cluster these individual antigenic residues into groups, and identify each of the groups as a candidate for a conformational B-cell epitope. The algorithm was evaluated on the three test data sets.

The algorithm’s *dist* parameter was set to range from 2Å to 18Å. The prediction results are summarized in Table 3. Previous literature has reported that the total number of residues per epitope ranges from about 10 to 20 [5], and we chose the distance 6 Å as the default distance threshold (*dist*) of this clustering algorithm.

**Table 3:** Statistical summary of the recommended conformational B-cell epitopes

Distance	N(cluster)	Maxlen	Minlen	Avelen	N(cluster)	Maxlen	Minlen	Avelen	N(cluster)	Maxlen	Minlen	Avelen
4	3.94	18.78	4.13	8.68	5.49	11.30	2.38	4.66	5.47	22.95	4.55	9.23
6	2.83	20.99	8.50	12.90	4.44	12.82	4.11	6.79	3.87	26.45	9.28	14.28
8	2.28	22.06	13.10	16.21	3.45	14.73	6.83	9.58	2.73	28.77	14.57	20.01
10	2.00	22.49	14.12	17.27	2.88	15.71	8.02	10.83	2.33	29.84	16.93	21.86
12	1.81	22.77	15.23	18.42	2.47	16.50	9.80	12.36	1.88	31.11	21.97	25.67
14	1.63	23.02	16.60	19.45	2.12	17.23	11.35	13.56	1.62	31.62	23.93	27.22
16	1.48	23.55	18.57	20.87	1.79	18.13	13.26	15.27	1.46	32.07	26.42	28.96
18	1.36	23.65	20.16	21.76	1.61	18.56	15.60	16.79	1.35	32.34	28.49	30.27

Three sets of results from the three test data sets (from left to right): ‘Ren92unbound’, ‘Exp106’ and ‘Exp165’. Each group includes columns of N(cluster), Maxlen, Minlen and Avelen. N(cluster) stands for the average number of epitopes on each antigen, Maxlen is the maximum length of residues of an epitope, and Minlen is the minimum length of residues of an epitope, Avelen indicates the average length of residues of an epitope

**Fig. 3:**
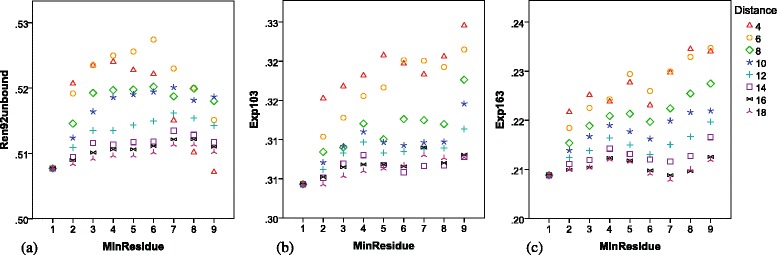
Performance by the recommendation algorithm under different parameters. (**a**) Ren92unbound, (**b**) Exp104, (**c**) Exp163. The *y-axis* shows the F-score. MinResidue refers to the minimum residues included in a recommended conformational epitope

Aggregated antigenic residues are more likely to constitute epitopes [21]. Based on this idea, we ranked the epitope candidates according to their number of residues. Figure 3 illustrates the change in performance when the recommendation level changed. After removing those isolated antigenic residues, SePre demonstrated further improvement, providing more useful and meaningful recommendations. On ‘Exp104’, the best F-score was 0.325 (*dist* = 4Å and *MinResidue* = 9); On ‘Exp163’, the best F-score was 0.235 (*dist* = 6Å and *MinResidue* = 9); and on ‘Ren92unbound’, the best F-score is 0.527 (*dist* = 6Å and *MinResidue* = 6). These results suggest that the algorithm’s default parameters should be 6Å for *dist* and 9 residues for *MinResidue*.

SePre uses the distance-based clustering algorithm with only antigen information to recommend conformational B-cell epitopes. Thus, it is unlikely to accurately locate each epitope to a specific antibody; especially, it cannot distinguish overlapped epitopes.

### Case studies

We present detailed prediction results on two case studies. The first case study was conducted on a glucose-dependent insulinotropic polypeptide receptor. It has a far kinship from any of the training sequences. The highest sequence similarity to the antigen chains in ‘Train190’ is only 19.5%, and only 9.6% to the ‘Liang19unbound’ data set. The epitope site of this antigen has been confirmed previously by several types of experimental methods, such as biological activity neutralization, surface plasmon resonance (SPR) dissociation and x-ray crystallography (IEDB Epitope ID: 194683).

**Fig. 4:**
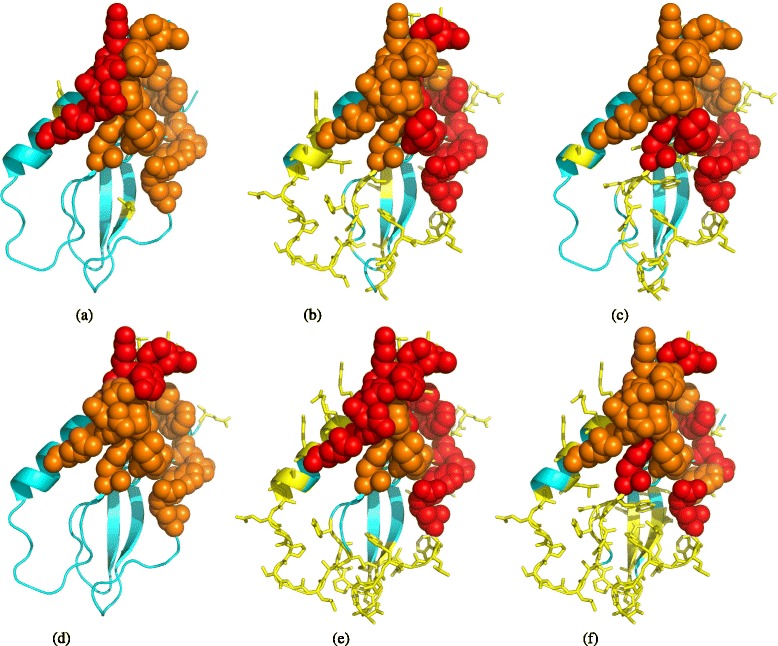
Prediction on a glucose-dependent insulinotropic polypeptide receptor (PDB ID: 2QKH, chain: A). (**a**) SePre, (**b**) Straightforward model, (**c**) CBTOPE, (**d**) DiscoTope 2.0, (**e**) ElliPro, (**f**) SEPPA 2.0. The epitope (IEDB epitope ID: 194683) is shown as *spheres*: the correctly predicted epitope residues (true positive) are colored in *red*, while the unrecognized epitope residues (false negative) are colored in *orange*. The non-epitope residues predicted as epitope (false positive) are shown as *yellow sticks*

SePre’s precision was the highest (0.667) for this epitope prediction in comparison to the other prediction methods (Fig. 4). SePre predicted six residues as antigenic residues: four of them (red spheres in Fig. 4 a) were true antigenic residues, and the remaining two were incorrectly identified as epitope residues (yellow sticks). By comparison, the straightforward model correctly identified nine of the 17 antigenic residues from amongst its total of 55 predicted antigenic residues. Its precision was only 0.164, much lower than ours.

The sequence-based method, CBTOPE, had a slightly lower F-score (0.320 compared to our 0.348). It correctly identified eight antigenic residues within its 33 positive predictions, and had a precision of 0.242. SePre also outperformed the three structure-based predictors. The predicted antigenic residues by ElliPro and SEPPA 2.0 dispersed substantially over the surface with a precision of only 0.210 and 0.111 respectively. DiscoTope 2.0 had the same recall with SePre, however, its precision was worse (0.444 compared to SePre’s 0.667).

The predicted antigenic residues were clustered into two conformational epitopes by Algorithm 1: one contained five nearby antigenic residues, and the other had only one antigenic residue (residue 101 in chain A). The larger one was recommended with a higher priority. The recommended five-residue conformational epitope consisted of nearby antigenic residues with only one false positive prediction. The precision was further improved to reach 0.80.

The second case study was conducted on an N9 neuraminidase of the influenza A virus (PDB ID: 1NCA, chain: A).

**Fig. 5:**
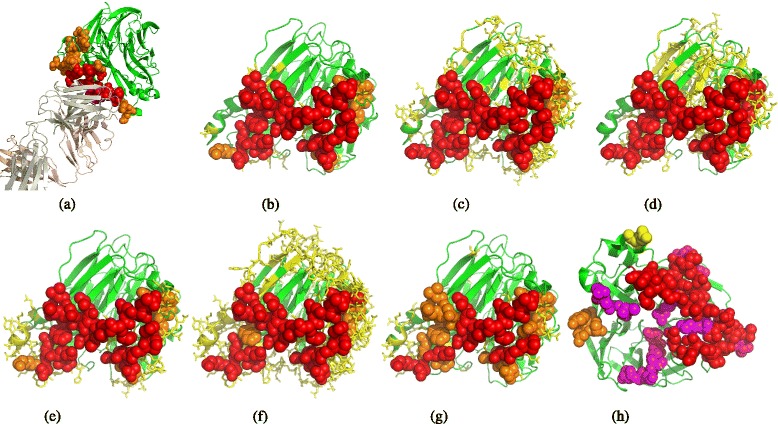
Prediction on an influenza A N9 neuraminidase antigen (PDB ID: 1NCA, chain: A). (**a**) Epitopes, (**b**) SePre, (**c**) Straightforward model (**d**) CBTOPE, (**e**) DiscoTope 2.0, (**f**) ElliPro, (**g**) SEPPA 2.0, (**h**) Conformational epitopes. The spheres represents the annotated epitope residues in **a**–**g**, and in **h** they represent the predicted epitope residues. The color mode for **b**–**g** are the same as Fig. 4. In **h**, three clusters are formed by Algorithm 1 and colored in *red* and *magenta*, *orange* and *yellow*. The largest cluster colored in *red* (annotated epitope residues) and *magenta* (other residues) is recommended with priority

Eight epitopes determined by various determination assays (IEDB ID: 77480-77483,77486-77489) can be mapped onto this antigen. These epitopes overlap, containing a total of 33 unique antigenic residues (Fig. 5).

SePre’s F-score was 0.650 with a recall of 0.788 and an excellent precision of 0.553. That is, of the 47 predicted antigenic residues, 26 are true antigenic residues. The straightforward model identified 28 true antigenic residues, with an improved recall of 0.848. Nevertheless, it made a total of 143 positive predictions. Its precision was only at a low level of 0.196, leading to an F-score of 0.318. This F-score was much lower than SePre’s 0.650.

The best performance of the other methods was made by DiscoTope 2.0 which had an F-score of 0.464. DiscoTope 2.0 also correctly identified 26 true antigenic residues (recall = 0.788), but its total of 53 false positive predictions was far greater than SePre’s total of 21, and thus its precision was only 0.329. The F-scores of CBTOPE, ElliPro, and SEPPA 2.0 were 0.292, 0.217 and 0.416, respectively.

Though our clustering algorithm, the predicted antigenic residues were grouped into three candidates for conformational epitopes (Fig. 5(f)). The first one (shown as red and magenta spheres) contains 42 antigenic residues, and the other two contain three (orange) and one (yellow) residues respectively. The largest one was preferentially recommended as a conformational epitope. As five false positive predictions (including the two small clusters and a buried residue) were removed from the recommendation and the 26 true predictions of antigenic residues were kept in the cluster. The prediction performance was further improved by the clustering algorithm.

## Discussion: where performance improvements are made in the staged heterogeneity learning model

This section presents the prediction performance by single propensities in the first stage, and describes how the performance is improved when the second stage is added. There are three important factors which can contribute to the excellence of the prediction performance: (i) choosing a good learning method on single propensities, (ii) choosing a good second stage learning method, and (iii) heterogenous learning (results already presented in “Prediction results by straightforward staged learning” section).

### The first-stage performance by single propensities

Propensities have been intensively used for epitope identification [1, 3, 25]. Epitopes also have strong preferences on certain propensities even for diversified antigens from different species [6]. In the first stage of our learning method, each propensity makes independent decisions on the probability of a residue being antigenic. Here, we describe the performance demonstrated by the various single propensities through LOOCV.

Table 4 summarises prediction performance by single propensity. The predictions were made using a random forest model and an SVM model under a LOOCV process on ‘Train190’. The 205 physico-chemical propensities were assessed separately (P1-P205). It can be seen that the sequence-predicted structural propensities ASA (P206) and secondary structure (P208), and the evolutionary propensity PSSM (P209) had a stable top-ranked predictive capability. By comparison, the static physico-chemical propensity based predictions varied greatly in their performance, especially in the SVM model. Another evolutionary propensity, conservation score (P207), had poor predictive power in both the random forest and SVM models. It is consistent with our previous results on the influenza hemagglutinin [26]: conservation score has a poor performance in identifying antigenic residues. Additionally, 85 antigens in ‘Train190’ have no conservation score information. This presents a significant obstacle for the conservation score based method. Given its poor performance and in-availability, conservation score was excluded from the construction of our learning method. Considering the average F-score on all the 209 propensities, the performance of the random forest model (0.252) was superior to SVM (0.219). Therefore, we selected the random forest approach, with the *mtry* parameter set as the window size, to train the sub-classifiers.

**Table 4:** Prediction performance of single propensities by LOOCV using Random Forest (column 2–5) and SVM (column 6–9)

Rank	Propensity	Recall	Precision	F-score	Propensity	Recall	Precision	F-score
Best1	P209	0.618	0.188	0.289	P206	0.720	0.177	0.284
Best2	P206	0.646	0.174	0.274	P209	0.630	0.179	0.279
Best3	P170	0.560	0.173	0.265	P208	0.724	0.167	0.271
Worst3	P122	0.362	0.165	0.227	P84	0.061	0.215	0.095
Worst2	P46	0.341	0.170	0.227	P166	0.090	0.100	0.095
Worst1	P44	0.283	0.169	0.212	P142	0.084	0.090	0.087
Average		0.537	0.165	0.252		0.407	0.163	0.219

The propensity 208 (secondary structure) ranks fourth in RF, and has a recall of 0.670, precision of 0.164, and F-score of 0.264. Propensity 207 (conservation score) ranks 201 and 100 in RF and SVM, and has an F-score of 0.239 and 0.235 respectively. Explanations to other important propensities: 209-PSSM, 206-ASA, 170-Optimized beta-structure-coil equilibrium constant (AAIndex ID: OOBM850101). AAIndex ID for worst propensities: 122-CHAM830107, 46-FAUJ880111, 44-FAUJ880112, 84-AURR980110, 166-OOBM770104, 142-GEOR030104. Antigens without a certain propensity were ignored in this table

From Table 4, we can observe that more powerful propensities generally have a remarkably higher recall, and a slightly higher precision than the less informative propensities. This suggests that epitope does have a strong preference on these propensities, and that a single propensity is not sufficient to accurately identify antigenic residues.

To reduce the potential impact of a few long antigen sequences on overall performance, an additional analysis was conducted by computing the average F-score on each complex. ASA and secondary structures were ranked as the top two propensities in both random forest and SVM methods, and achieved an average F-score of more than 0.395. PSSM had an average F-score of 0.378 (RF) and 0.362 (SVM), while the conservation score had an average F-score of 0.283 (RF) and 0.268 (SVM), respectively.

### Why need the second stage to learn the intrinsic complementarity of propensities and which learning method is appropriate

Since various propensities contribute to binding affinity, the collective synergy of the propensities should be incorporated in any prediction model. Existing studies have investigated several ways to integrate the propensity complementarity, like linear combination [17], re-sampling and voting [20], or data mining methods [19].

Here, we compare the performance of several propensity integration methods, including simple voting methods methods (ranked and exhaustive), data mining methods (SVM and random forest), Bayesian methods (Naïve Bayesian and Bayesian network) and tree algorithms (decision tree and regression tree). These experiments were performed on the data derived from the first-stage prediction probabilities by LOOCV on the training data set ‘Train190’. A dummy value or vote was assigned when a propensity value was not available. The performance results are listed in Table 5.

**Table 5:** Performance by various propensity integration methods

Group	Methods	Propensities	Recall	Precision	Fscore
Voting	Ranked voting^a^	209	0.650	0.191	0.295
	Exhaustive voting	5 groups	0.656	0.191	0.295
General	SVM	5 groups	0.614	0.181	0.279
	Random forest	5 groups	0.600	0.186	0.284
Bayesian	Naïve Bayesian	5 groups	0.686	0.193	0.301
	Bayesian network	5 groups	0.775	0.173	0.283
Tree	Decision tree	5 groups	0.675	0.196	0.304
	Regression tree	5 groups	0.655	0.194	0.299
General	SVM	209	0.594	0.191	0.289
	Random forest	209	0.639	0.191	0.294
Bayesian	Naïve Bayesian	209	0.633	0.198	0.301
	Bayesian network	209	0.586	0.170	0.264
Tree	Decision tree	209	0.633	0.198	0.301

The parameters used in these models are tuned to realize an optimal performance. The best ranked voting was achieved by propensity PSSM (P209), ASA (P206) and optimized beta-structure-coil equilibrium constant (P170, AAIndex ID: OOBM850101). The best exhaustive voting results was realized by a combination of PSSM, physico-chemical propensities and ASA

^a^Due to the complexity of exhaustive voting algorithm, only five groups of propensities were used. Regression tree constructed on 209 propensities required too much computation time, and no results were obtained

The ranked voting algorithm was carried out on all the 209 propensities. Voting on the three propensities PSSM, ASA and the optimized beta-structure-coil equilibrium constant achieved optimal performance: the best F-score was 0.295, and the recall and precision were 0.650 and 0.191 respectively. The exhaustive voting algorithm was performed on the five groups of propensities, and the best performance was achieved by a combination of PSSM, ASA and all the physico-chemical propensities. The best F-score was also 0.295, while the recall (0.656) was slightly better than the ranked voting algorithm (0.650), indicating that the incorporation of the other 204 physico-chemical propensities has little contribution to the overall performance.

For the data mining methods with five groups of propensities, SVM and random forest did not improve prediction performance over single propensity based sub-classifiers, even after parameter optimizations. The best F-score was only 0.284 (by random forest), which was not better than only using a PSSM profile (0.289). Yao et al. mentioned that sophisticated data mining methods are more suitable for larger numbers of propensities [30]. These data mining methods were thus further applied to all the 209 propensities, to fully exploit the potential of these data mining algorithms. As shown in the bottom rows of table, these data mining methods achieved improved performance over the five-propensity counterparts with a better F-score of 0.294 (random forest model).

By comparison, simpler data mining methods, such as decision tree and Naïve Bayesian, seemed to yield better performance with both higher recall and greater precision. The decision tree algorithm, based on five groups of propensities, performed best with an F-score of 0.304, a recall of 0.675 and a precision of 0.196.

The reason for the varied results of different propensity integration methods is possibly attributed to the nature of the problem. The collective synergy of propensities in epitopes should not be a complicated problem, but heterogeneity exists among samples. Voting algorithms oversimplify the problem, and fail to consider the different contributions of propensities in epitope identification. Advanced data mining methods, such as SVM and random forest, overfit the training data, and achieve very little improvement on heterogeneous samples. The simple data mining methods, like decision tree and Naïve Bayesian, however, make a tradeoff between complexity and overfitting, which provides a tremendous value to quality heterogeneity learning.

## Conclusions

In this paper, we proposed a staged heterogeneity learning algorithm to deal with the high complexity of heterogeneity in antibody-antigen interactions for accurate conformational B-cell epitope prediction. The prediction model first learns the complicated epitope patterns of each type of propensities, and then learns the complementarity of propensities on a small guided heterogeneous data set. With only sequence information, our proposed model outperformed the state-of-the-art sequence-based and structure-based prediction methods on both heterogeneous and homologous data sets. We also investigated how these dramatic performance improvements were made, by assessing the performance of a straightforward staged learning model, single-propensity based sub-classifiers, and various propensity integration methods. A clustering algorithm was also designed to make recommendations for conformational B-cell epitopes from the predicted antigenic residues to provide more applicable results for vaccine design and development.
